# Multiple myeloma, a quintessential malignant disease of aging: a geroscience perspective on pathogenesis and treatment

**DOI:** 10.1007/s11357-022-00698-x

**Published:** 2022-12-12

**Authors:** Veronika S. Urban, Andrea Cegledi, Gabor Mikala

**Affiliations:** 1 Department of Morphology and Physiology, Faculty of Health Sciences, Semmelweis University, Budapest, Hungary; 2 Department of Hematology and Stem Cell Transplantation, South Pest Central Hospital–National Institute for Hematology and Infectious Diseases, Budapest, Hungary

**Keywords:** Multiple myeloma, aging, Gammopathy, Frailty, Vulnerable patient

## Abstract

Multiple myeloma (MM) is an incurable plasma cell malignancy, which is predominantly a disease of older adults (the median age at diagnosis is 70 years). The slow progression from asymptomatic stages and the late-onset of MM suggest fundamental differences compared to many other hematopoietic system-related malignancies. The concept discussed in this review is that age-related changes at the level of terminally differentiated plasma cells act as the main risk factors for the development of MM. Epigenetic and genetic changes that characterize both MM development and normal aging are highlighted. The relationships between cellular aging processes, genetic mosaicism in plasma cells, and risk for MM and the stochastic processes contributing to clonal selection and expansion of mutated plasma cells are investigated. In line with the DNA damage accumulation theory of aging, in this review, the evolution of monoclonal gammopathy to symptomatic MM is considered. Therapeutic consequences of age-dependent comorbidities that lead to frailty and have fundamental influence on treatment outcome are described. The importance of considering geriatric states when planning the life-long treatment course of an elderly MM patient in order to achieve maximal therapeutic benefit is emphasized.

## Introduction

Multiple myeloma (MM) accounts for about 1% of all cancers and approximately 10% of all hematologic malignancies. Aging is considered one of the most significant risk factors for various malignant diseases. MM excels in this regard, as it affects predominantly older people. It is diagnosed on average at the age of 70, and only a tiny proportion (less than 2%) of patients with MM are less than 40 years old [1]. Males have a 20% higher chance of a MM diagnosis than females [2]. Excess body fat is known to increase the lifetime risk of developing MM [3].

MM is considered a rare disease, but its global incidence increased by 126% from 1990 to 2016 [4], probably due to the worldwide aging population. In fact, it is the most common malignancy that originates in the bone marrow. In MM, the origin of the cells that serve as the starting point for the malignancy is not the bone marrow stem or progenitor cells, like in most other hematopoietic malignancies, but terminally differentiated, mature B cells in the germinal centers of secondary lymphoid tissues [5]. Symptomatic MM is characterized by the clonal expansion of terminally differentiated, antibody-producing plasma cells, progenies of a malignantly transformed B cell. Plasma cells in MM produce non-functional monoclonal antibodies (M proteins) that result in elevated serum levels in most patients, termed a monoclonal gammopathy. Malignant clones later tend to colonize the bone marrow but also may cause damage to soft tissues in the form of plasmacytomas. The diagnosis of MM is primarily based on an elevated clonal plasma cell count (or plasmacytoma) and nowadays less importantly of the M protein and disease defining CRAB criteria (hypercalcemia, renal insufficiency, anemia, and osteolytic bone destruction). In most cases, gammopathies very slowly develop from the asymptomatic forms [6] towards the manifest MM [7]. More recently, the SLiM diagnostic criteria were also established for defining myeloma: smoldering myeloma with more than 60% plasma cells, with more than 100 for free light chain ratios, and/or more than one focal lesion on MRI scans is also termed as MM that requires therapy.

In this review, the stochastic genetic, epigenetic, and cellular processes involved in clonal selection and expansion of mutated plasma cells are presented from a geroscience perspective, highlighting the role of basic mechanisms of aging in the pathogenesis of MM. The goal of therapy—especially in the elderly—should be to slow down disease progression, achieve symptomatic relief, possibly a remission, and provide the best possible quality of life, considering patients’ older age and comorbidities.

The biological and clinical heterogeneity observed in MM results in variable responses to treatment and outcome. Therefore, while tailoring the treatment, predictive and prognostic biological markers, as well as geriatric/frailty assessments, are to be considered according to the recommendation of the International Myeloma Working Group (IMWG) [8]. Here, an overview of the clinical practice and experiences as well as recommendations for elderly, unfit, and frail patients are provided.

## Diverse mechanisms underlying the long-lasting pathogenesis of symptomatic MM

Epigenetic changes and genetic mutations with structural and numerical chromosome aberrations that characterize MM may develop asymptomatically for decades. Prolonged disease progression and branching evolution lead to clonal heterogeneity of malignant cells in different patients [8]. Additionally, the disorder has different cytogenetic backgrounds despite similar clinical diagnostic criteria [9]. There are no two MM patients alike, though certain biologic subclasses of the disease are clearly distinguishable. Due to its complexity and heterogeneity, our understanding has been developing at least as slowly and gradually as the MM itself. The IMWG has differentiated asymptomatic plasma cell disorders from asymptomatic MM in their 2009 recommendations for differential diagnosis [10]. According to more recent updates by Rajkumar [11], subcategories of these asymptomatic stages are non-IgM as well as IgM monoclonal gammopathies of undetermined significance (MGUS and IgM MGUS), light-chain MGUS, and smoldering multiple myeloma (SMM). These phenotypes lack myeloma-defining events (MDE), such as end-organ damage or amyloidosis. However, patients affected have an abnormally higher rate of monoclonal B cells in their bone marrow (MGUS, SMM) or elevated serum and urinary monoclonal protein that is a class of IgA or IgG (in MGUS), IgM (in IgM MGUS), or light chain (in light-chain MGUS). The asymptomatic MGUS and SMM disorders tend to develop into MM at different rates and speeds, depending on their genetic background. Interestingly, the MGUS is known to be present in approximately more than 3% in the population above the age of 50 [12]. MGUS progresses to multiple myeloma or related malignancies at a steady rate of 1% per year [13], meaning that MGUS diagnosis may precede the development of MM by more than 10 years [14]. SMM is considered a more advanced stage of the developing malignancy, and it tends to develop into symptomatic MM at a rate ten times higher than seen in MGUS, especially in the first 2 to 5 years after its diagnosis. SMM may also have early active forms depending on genetic changes and require closer follow-up [15].

Multiple myeloma was initially termed “multiple” to distinguish it from plasmacytoma, a solitary tumor of a bone or an organ/tissue. Solitary plasmacytoma (SP) may develop further into a solitary plasmacytoma with minimal marrow involvement (SPMMI). Both SP and SPMMI are characterized by monoclonal plasma cell contribution confirmed by biopsy evidence [16]. By contrast, MM typically has multiple foci of the clonal proliferation of malignant plasma cells in the bone marrow, and these form multiple osteolytic lesions in the involved bones, most commonly in the vertebrae, skull, ribs, pelvis, and the proximal long bones [10]. SP has a much better prognosis than MM. However, symptomatic MM is developing in almost 50% of patients with SP of bone. This unfavorable progression may take as long as 15 years [17].

### MM in companion animals

Veterinary medicine offers a unique insight into the biology of age-associated diseases, including malignancies. In the field of geroscience, dogs are considered highly interesting models to understand biological and environmental factors that influence aging, age-related pathologies, and longevity. Unlike laboratory rodents, companion dogs and humans are exposed to similar environmental conditions. There is strong evidence that many chronic conditions that are manifested in older humans (e.g., obesity, chronic inflammatory diseases, diabetes mellitus), and which are associated with comorbidities, are also present in companion dogs and are associated with similarly high levels of comorbidities [18, 19]. In both dogs and humans, malignancies are leading causes of death, and age trajectories of deaths from malignant diseases are almost identical between the two species [18]. Multiple myeloma also develops in older dogs, and it represents approximately 1% of all canine tumors [20–29]. An analysis of myeloma in 156 cases in companion dogs reported diagnosis at the median age of 10.7 years [20] (37% of maximum lifespan potential in *Canis familiaris* [30] and dogs are generally considered old after 10 years of age). Thus, multiple myeloma is an age-related malignancy in dogs, similarly to humans (the median age of humans with multiple myeloma at time of diagnosis is ~70 year, which is 57% of the maximum lifespan potential of the species). Dogs are also considered powerful models of genetic determinants of successful aging [31, 32], as the aging profiles of different dog breeds vary according to their adult size: smaller dogs often live for over 15–16 years, medium and large size dogs have a lifespan typically of 10 to 13 years, and some giant dog breeds such as mastiffs often live only for 7 to 8 years. It is interesting to consider that certain relatively shorter-lived dog breeds seem to be more susceptible to developing plasma cell tumors, including German shepherds (median lifespan: ~10.3 years) [33].

Cats also develop myeloma at old ages [34–45]. A detailed analysis of myeloma cases in sixteen cats reported a median age of 14.0 years (47% of maximum lifespan potential in *Felis catus domesticus* [30] at time of diagnosis [46]. In contrast to other hematological malignancies (e.g., lymphoma) in cats, there is no evidence that would suggest that infection with oncogenic viruses (e.g., feline immunodeficiency virus, feline leukemia virus) is causally linked to the pathogenesis of feline cases of multiple myeloma [47]. The available evidence suggests that in contrast to the model of primary intramedullary neoplastic transformation explaining the pathogenesis of multiple myeloma in humans, in felines primary extramedullary neoplastic transformation (plasmacytoma) is more common [48].

Many characteristics of myeloma are similar in humans and companion animals. Multiple myeloma in companion animals appears to exhibit an immunoglobulin isotype distribution that is similar to human cases: IgG and IgA gammopathies are the most common in both people and companion animals (dogs and cats). Both dogs and cats exhibit evidence of skeletal lesions, similarly to humans.

Taken together, veterinary medicine may offer valuable insight into the gero-oncological aspects of the pathogenesis of MM. Future studies are also warranted to investigate the similarities of risk factors for myeloma in humans and companion animals, including the role of obesity and exposure to environmental toxicants (e.g., the presence of benzene in food consumed by companion animals; see below).

## Potential roles of fundamental cellular and molecular mechanisms of aging in the pathogenesis of MM

Geroscience research in the past 2 decades has demonstrated that the rate of organismal aging is controlled by evolutionarily conserved genetic pathways and biochemical processes [49]. By definition, the pathogenesis of all age-related diseases, including that of MM, must involve these processes and pathways. In the following section, the potential roles of fundamental cellular and molecular mechanisms of aging in the pathogenesis of MM are discussed.

### Reactive oxygen species, DNA damage, and mutation accumulation

Aging is characterized by increased production of reactive oxygen species (ROS) and increased susceptibility to ROS-mediated DNA damage. Importantly, DNA damage mediated by ROS (either produced by cellular sources or generated by exogenous factors, e.g., UV radiation, tobacco smoke constituents, and other environmental toxicants) plays a central role in the accumulation of different mutations in B cells, ranging from a single nucleotide divergence to the broken chromosomes and ploidy changes [50], promoting the pathogenesis of MM.

There is strong evidence that in addition to increased production of ROS, aging impairs pathways underlying cellular resilience to oxidative stressors. In young cells, evolutionarily conserved antioxidative stress resilience pathways maintain cellular reduction-oxidation homeostasis through transcriptional regulation of key cytoprotective genes encoding antioxidant enzymes, pro-survival factors, anti-inflammatory, and macromolecular damage repair pathways. A critical mechanism by which aging may exacerbate the induction of DNA damage in plasma cells and thereby the pathogenesis of MM involves an age-related impairment of oxidative stress resilience pathways [51–55] and, consequentially, increased propensity for ROS-mediated alterations to the DNA.

One of the most abundant spontaneous hydrolytic reactions also points to the close interaction between mutations and epigenetic impairment (see below). Cytosine with an epigenetic mark, i.e., 5-methylcytosine, tends to easily lose an amino group and therefore easily be converted to thymine. As this spontaneous mutation primarily occurs at methylated CpG islands [56], we can speculate that quiescent (e.g., memory B cells) or senescent cells are particularly vulnerable to mutations at the loci of cell cycle regulation and, therefore, can be the source of a malignant clone with time. Every tissue accumulates mutations at a roughly constant mutation rate throughout life; the estimated rate is 13–44 mutations per genome per year [57]. Therefore, with age, genetic mosaicism seems to be inevitable, and it is only a question of time when the fitness of a clone leads to clonal expansion to the detriment of others [57]. Stochastic processes may produce malignant, benign, or natural/functional clones or senescent cells.

#### Role of DNA damage induced by environmental toxicants in the pathogenesis of MM

Several toxic chemicals to which humans are exposed have been causally linked to the pathogenesis of MM through their ability to induce oxidative stress and/or DNA damage. An important example is benzene which is a natural constituent of crude oil and is one of the most widely used chemicals in developed countries. Benzene is highly toxic and is used to manufacture resins, adhesives, nylon fibers, plastics, rubbers, lubricants, dyes, detergents, drugs, explosives, and pesticides. There are many studies linking exposure to benzene to myeloma [58–63]. Benzene is present in petrol, and inhalation of benzene with petrol fumes (e.g., taxicab drivers [64], workers in gasoline stations [65], and refineries) results in a significantly increased risk for malignancies. Exposure to the herbicide glyphosate (Roundup^®^) has also been linked to the genesis of myeloma and other malignancies [66, 67], and on the basis on findings of epidemiological studies and preclinical investigations, the World Health Organization’s International Agency for Research on Cancer (IARC) classified it as “probably carcinogenic in humans” (category 2A). Although some controversies exist regarding the methodologies of epidemiological studies [68, 69], exposure of laboratory rodents under controlled conditions results in induction of myeloma. Age-related decline in cellular stress resilience likely renders aged B cells more susceptible to environmental toxicants.

#### Possible mechanisms contributing to the increased susceptibility of B cells to DNA damage and malignant transformation

During normal B cell maturation and development to plasma cells in germinal centers, the several developmental steps—reviewed in detail elsewhere [70]—make these cells a particularly good target for mutations, increasing the risk of potential malignant transformation. Firstly, genetically inherent steps of somatic hypermutations are needed for the most effective B cell receptor and antibody-producing clones. Somatic hypermutations involve a step when cytosine deamination occurs with enzymatic help (activation-induced cytidine deaminase, AID) at genomic regions coding variable regions of immunoglobulins. This way, germinal centers are the places of “officially” promoted mutations. Enormous cellular and intracellular networks [70] are responsible for the mutations and the proper clone selection via epigenetic changes; the more steps needed, the more mistakes may occur. Secondly, multiple cell divisions are needed that lead to proliferation and survival vs. extinction of suitable vs. not suitable clones for effective humoral immune response vs. autoimmune responses. For clonal selection, clones are needed. The single-stranded DNA during replication is more vulnerable to mutations. Thirdly, to preserve a long-term humoral memory, quiescent B cells are preserved with an arrested cell cycle for a long time, necessarily with hypermethylated regions of their genome at cell cycle regulation. We speculate that, similarly to senescent cells, memory B cells, due to the vulnerability of the hypermethylated regions, may become malignant, depending on other mutations accumulated. Due to the aforementioned factors, B cells are susceptible to DNA damage, which may lead to further structural and numerical chromosomal mutations. B cells divide in germinal centers, but healthy plasma cells do not. Due to cascading mutational events that start in B cells, a plasma cell clone may proliferate and gain further mutations. The monoclonal source of a dysfunctional antibody is detectable in gammopathies [5].

### Cellular senescence

Cellular senescence, an evolutionarily conserved, DNA damage-associated cellular stress response, is a critical biological process underlying aging [71]. Several molecular stressors associated with aging, including oxidative and nitrative stress, mitochondrial dysfunction, inflammatory cytokines, and replicative exhaustion/telomere attrition, may trigger cellular senescence [72–74]. Cellular senescence results in stable growth arrest whereby cells stop proliferating in spite of the presence of growth signals. In order to escape apoptosis in replicative arrest, senescent cells inactivate p53 and other proteins with apoptosis-induction roles in case of DNA damage [74]. This may act to facilitate malignant transformation in senescent and pre-senescent cells [75]. Other mechanisms by which senescent cells may indirectly promote the pathogenesis of MM involve changes in the cellular microenvironment. Senescent cells have intense metabolism and exhibit a pro-inflammatory senescence-associated secretory phenotype (SASP). Chemokines, pro-inflammatory cytokines, growth factors, and matrix proteases secreted by senescent cells likely contribute to the genesis of a wide range of age-related diseases and may play a critical role in the development of a growth promoting tumor microenvironment that influences the growth, invasion, and metastasis of transformed cells [71, 76]. Senescent cells survive for a prolonged time, occupying up to more than 10 percent of aging tissues [77] that may become gradually dysfunctional.

### Epigenetic dysregulation: changes in DNA methylation

Epigenetic alterations are important hallmarks of aging [49] and play an important role in the pathogenesis of MM [78–99]. In the field of geroscience, there is an important distinction between biological age (determined based on alterations in the epigenome and consequential phenotypic and functional changes) and chronological age [100–102]. Biological age acceleration (which indicates unsuccessful/unhealthy aging due to lifestyle factors, among other) was shown to associate with DNA methylation-based cancer risk factors [103–106]. Future studies should determine how age acceleration, via altering the epigenome, promotes the pathogenesis of MM.

As shown in the differentiation studies, DNA hypermethylation at CpG islands in a gene’s promoter region can switch off its transcription, and hypomethylation of these areas switches them on, depending on the cell’s fate during development. There is a global DNA hypomethylation in malignant cells, including MM cells [107]. Overall hypomethylation leads to genomic instability, e.g., release of the transposable elements, which lead to mutations and disease progression [107], [108]. Hypermethylation of distinct genes also occurs in malignant cells as well as aged cells. In myeloma cells, genes typically affected are tumor suppressor genes [109], [110], leading to the loss of cell cycle control, and genes involved in stress resilience, including DNA repair genes [92, 111]. Epigenetic aberrations have also been causally linked to chemoresistance in MM [112]. In senescent cells, global hypomethylation is accompanied by hypermethylation of the senescent-associated heterochromatin foci (SAHFs). The SAHFs are at the loci of proliferative genes such as E2F target genes, responsible for initiating the G1 phase of the cell cycle. This way, senescent cells may live with replicative arrest for a long time. Epigenetic changes that affect cell cycle regulation may lead directly (via silencing of tumor suppressor genes) or indirectly (via regulation of cellular senescence) to malignancy. In this regard, see also possible prevention strategies later. Due to MM’s late-onset, we have no reason to doubt that myeloma development lacks the effect of senescent cells or that the origin of the malignant clone is not a long-lived memory B cell with senescence-like properties.

### Epigenetic dysregulation: histone modifications

In addition to DNA methylation, posttranslational histone modifications also have an important role in regulation of chromatin structure and thereby in the transcriptional regulation of pathways that modulate aging [113–115]. Histone proteins form a complex with DNA to compile the nucleosomes consisting of 2 subunits of each core histone (H2A, H2B, H3, and H4). Changes in the abundance of core histones and the ratio of different histone variants may also affect genomic stability both in aging cells and malignant clones in MM. Reduced histone protein synthesis with aging results in a loss of histones and changed nucleosome positioning on particular DNA sequences. This age-related decrease in nucleosome abundance may contribute to dysregulation of gene expression and increased genomic instability, including increased DNA breaks, translocations, and increases in retrotransposons and the insertion of mitochondrial DNA into nuclear DNA [116], all of which may promote the pathogenesis of MM. During aging in mammals, the abundance of non-canonical histone protein variants increases [117], which may also affect the histone methylation patterns. Histone variants may be replaced at any stage of the cell cycle [118]; thus, dynamic epigenetic changes may occur even in non-dividing cells, potentially contributing to cancer formation. It can be hypothesized that similar processes may occur during aging of memory B cells with an arrested cell cycle, which may lead to multiple cytogenetic changes. Previous studies identified genes encoding histones and diverse histone modifiers as targets of somatic mutation in MM [119]. Pawlyn et al. found mutations of the HIST1H1B-E gene family encoding H1 histone in 6% of 463 MM patients and, with lower frequency, mutations in genes encoding core histone proteins [120].

The N-terminal domains of the core histone proteins (histone tails) are the sites of posttranslational modifications, including methylation, acetylation, phosphorylation, ubiquitination, and sumoylation. These histone marks present an additional layer of epigenetic information that affects the chromatin structure and, consequently, gene transcription, DNA replication, and repair [121]. There are distinct changes in histone marks in aging cells. A global increase in active histone marks such as H3K4me3 and H4K16ac and a decrease in repressive marks such as H3K9me3 and H3K27me3 associate with aging. These changes further increase the instability and vulnerability of the genome. Not surprisingly, aberrant histone posttranslational modification profiles are also linked to tumorigenesis [122]. The case with histone code is complex with many site-, cell-, and tumor-type-specific changes in aging and MM [79, 119]. Dysregulation of histone modifiers (e.g., histone methyltransferases, acetyltransferases, demethylases, deacetylases) and their associated histone mark profiles in MM pathogenesis were recently reviewed [107].

The interaction between epigenetic alterations associated with aging and the pathogenesis of cancer is subject to intensive research. Sirtuins (Sirt 1-7) are evolutionarily conserved histone modifiers, which function as NAD+-dependent histone deacetylases. Sirt proteins regulate essential metabolic pathways and are involved in cell survival, senescence, proliferation, apoptosis, DNA repair, cell metabolism, and caloric restriction[123–138]. In particular, Sirt 6 seems to play a role in MM as its high expression is related to poor prognosis [139]. In contrast, SIRT2 and SIRT3 expression is reduced in MM patients as compared to healthy controls [140]. Sirtuins are considered potential targets for treating neurodegenerative diseases, cardiovascular diseases, cancer, and aging [133–138, 141]. The role of SIRT1 activators and that of other histone modifiers in the pathogenesis and treatment of MM is a focus of intensive investigations [120, 142–144].

### Post-transcriptional regulation of gene expression

Post-transcriptional regulation of gene expression represents an additional layer of epigenetic regulation. Long non-coding RNAs (lncRNAs) are RNA transcripts more than 200 nucleotides long that are not translated into protein, which are involved in both in transcriptional regulation and post-transcriptional regulation of gene expression. There is growing evidence that lncRNA-mediated regulation of gene expression plays a role in modulation of cellular aging processes [145–149]. Importantly, lncRNAs are useful biomarkers for the diagnosis and prognosis of MM [150]. lncRNAs have been causally linked to dysregulation of cell proliferation, tumor growth, and drug resistance in MM [151–160].

MicroRNAs (miRNAs) are small single-stranded non-coding RNAs (containing about 22 nucleotides), whose functions include RNA silencing and post-transcriptional regulation of gene expression [161–164]. Dysregulation of miRNA expression was shown to contribute to the genesis of aging phenotypes in various organs [165–173] including cells of the immune system [174, 175]. miRNAs play important roles in the generation and differentiation of B cells [176–178]. Importantly, dysregulation of miRNA expression has been demonstrated in MM, which likely plays a multifaceted role in the pathogenesis of the disease [179–186].

### Dysregulation of proteostasis

Normal proteostasis, the homeostatic regulation of the functional proteome, is essential for the maintenance of youthful organ function. Age-related changes of the proteostasis network underlie the altered biogenesis, folding, trafficking, and degradation of proteins in aging cells and tissues and contribute to the pathogenesis of a wide array of age-related diseases [187–198].

Survival and proliferation of myeloma cells, which unusually produce large amounts of abnormal immunoglobulins, critically depend on a normal proteostasis. Factors that overwhelm the proteasome (e.g., proteasome inhibitors, such as bortezomib or heat shock [199] result in increased cellular stress and promote apoptosis in myeloma cells.

### Nutrient sensing pathways

Nutrient-sensing pathways, including GH/IGF-1 pathway, mammalian target of rapamycin (mTOR), sirtuins, and AMP-activated protein kinase (AMPK) signaling, regulate organismal and cellular metabolism and modulate cellular processes of aging [49, 200, 201]. These pathways also play critical roles in the regulation of metabolism and survival of malignant cells, including MM cells. IGF-1 signaling promotes myeloma cell survival and progression [202–207], whereas inhibition of IGF-1 signaling promotes the pro-apoptotic effects of chemotherapeutic drugs in MM. mTOR regulates cellular protein synthesis, autophagy, cell growth, and proliferation as well as cell survival and thereby modulates aging processes and lifespan regulation. In myeloma cells, mTOR signaling plays an important pro-survival role, and pharmacological inhibition of its activity was shown to exert significant, therapeutically relevant cytotoxic effects [208–214]. AMPK plays a central role in regulation of cellular energy homeostasis, by activating glucose and fatty acid uptake and metabolism. Activation of AMPK was shown to delay aging and extend lifespan in models of aging. Metformin is a pharmacological activator of AMPK, which was shown to extend lifespan in rodent models, and its anti-aging activity is to be tested in clinical studies as well [215–217]. Importantly, metformin was shown to exert significant anti-myeloma effects in preclinical studies and is also being considered for myeloma treatment in human patients [218–224].

### Age-related sterile inflammation and immunosenescence

Aging per se results in chronic low-grade sterile inflammation (termed “inflammaging”) in multiple tissues due to age-related phenotypic changes in a number of cell types, including cells of the cardiovascular system, adipocytes, fibroblasts, and immune cells [225]. The highly proinflammatory SASP of senescent cells is an important contributing factor [226–229]. Inflammaging results in increased levels of pro-inflammatory mediators in the circulation and tissues and is a strong risk factor for multiple age-associated diseases. Various lifestyle factors, including obesity associated with consumption of an unhealthy diet, exacerbate age-related inflammation. Obesity in older adults is associated with a heightened state of inflammation in the white adipose tissue and with a consequential increase in circulating levels of pro-inflammatory cytokines [230–232]. Increased systemic inflammation in obese older subjects is known to promote the pathogenesis of MM [233–235]. Other obesity-associated cancers may also be present in obese older MM patients.

Advanced aging associates with immunosenescence, which is characterized by immune dysfunction increasing susceptibility to infectious pathogens, compromising vaccine responses and promoting the development of autoimmune diseases and malignant tumors in older adults. Immunosenescence mainly affects the adaptive immune responses by decreasing lymphopoiesis, while myelopoiesis is maintained. Immunosenescence has also been causally linked to the pathogenesis of frailty and age-related cardiovascular diseases and degenerative diseases [236]. The heightened state of inflammation in older adults combined with an age-related decline in tumor immune surveillance favors the genesis of MM[236].

### Clonal hematopoiesis

Clonal hematopoiesis of indeterminate potential (CHIP) is a newly recognized phenomenon in older adults. There is growing evidence that somatic mutations are manifested in blood cells and/or cells of the bone marrow in 10% of adults aged 70 to 80 [237–240]. In CHIP, hematopoietic stem cells with somatic mutations modulate hematopoietic lineage potential by a mechanism of clonal expansion. CHIP is associated with an increased risk for hematologic malignancies and cardiovascular diseases [237–248]. It also associates with adverse outcomes in patients with other advanced malignancies [249]. In MM the presence of CHIP in bone marrow is also associated with poor prognosis and development of second hematopoietic malignancies [250].

## Importance of cytogenetic heterogeneity for the progression of MM

There are no two MM patients alike. The genetic background of the same disease phenotype in MM varies greatly, which also influences the progression of the disease. According to Rajkumar’s update [11], the clonal plasma cell population of MGUS or SMM cells are primarily gaining multiple odd-numbered chromosomes or immunoglobulin heavy chain translocations. Trisomies are present in the plasma cells obtained from the bone marrow in approx. 40% of the MM cases, termed trisomic (or hyperdiploid) MM. Almost all the rest of the myeloma patients have a translocation involving the immunoglobulin heavy chain (IgH) locus at chromosome 14q32; their condition is commonly termed as IgH-translocated MM. Both numerical and structural chromosomal changes, i.e., trisomies and IgH translocations, can be present in rare cases up to 15%. Further cytogenetic abnormalities may arise later in the progression of the MGUS/SMM condition or MM [11]. These typically include gains or losses of short or long arms of certain chromosomes e.g., gain(1q), del(1p), del(17p), del(13); *RAS* mutations, and secondary translocations [251].

IgH locus at 14q32 is transcriptionally active in B cells; therefore, patients with IgH translocation may have oncogenes translocated to the 14q32 region, which may lead to their enhanced expression. There are several known reciprocal translocations of 14q32 with other chromosomes, including t(4;14) and t(11;14) translocations (30% of patients with MM) and the less common t(14;16), t(6;14), t(8;14), and t(14;20) translocations. For example, the t(4;14) translocation can be associated with upregulation of the fibroblast growth factor receptor 3 (FGFR3) and the myeloma SET domain protein (MMSET). Affected patients show an overall poor prognosis and therapeutic resistance. The reciprocal translocations of the IgH allele at 14q32 with other oncogenes such as D types of cyclins, Maf family members, or c-MYC also affect the disease course [252]. In addition, patients with t(4;14) translocation, del(17p), and gain(1q) are at a higher risk of progression from MGUS or SMM to MM [253].

The effect of any cytogenetic abnormality varies depending on the stage of the disease. Complexity, variability, and randomness all suggest that there is essentially an aging process in B-cells resulting in a vulnerable epi/genomic state and, finally, the propagation of a malignant clone at work in MM.

## Treatment aspects of MM in older adults

Therapy of MM has been revolutionized in the last 2 decades with the registration and availability of multiple highly effective novel agents, such as immunomodulatory agents, proteasome inhibitors, monoclonal antibodies, and more recently, CAR-T cells and bispecific antibodies. Despite these advances, effective treatment of MM in unfit and frail older adults is still a challenge.

Frailty is a functional term representing the multisystem decrease in physiological functions that leads to increased vulnerability, which, in return, results in increased morbidity and mortality during cancer therapy. Since MM is a disease that affects predominantly older adults, frailty may influence the therapy of up to two-thirds of MM patients. These patients are frequently designated as “not eligible for autologous stem cell transplantation,” which is a highly effective standard procedure for younger patients. Yet, this classification may be entirely misleading: multiple different cohorts of older MM patients exist. Therefore, it is paramount to properly address frailty and adjust anti-myeloma therapy accordingly to obtain a maximal therapeutic benefit. As elderly and frail patients are less likely to participate in clinical trials, the evidence of their proper therapy is of lower grade, mainly based on results of fit elderly patients that did participate and on the expert opinions of recommended dose adjustments.

In order to properly identify myeloma patients who are at increased risk of therapy-related toxicity, clinical frailty scores were established. These scores aim to help predict survival, likely adverse event rate, and treatment tolerability. In the field of myeloma, the most accepted frailty scoring system is the International Myeloma Working Group (IMWG) frailty score [254]. It uses age, the Katz Activity of Daily Living (ADL), the Lawton Instrumental Activity of Daily Living (IADL), and the Charlson Comorbidity Index (CCI), as shown in Table 1 (and a convenient web-based tool is available at http://www.myelomafrailtyscorecalculator.net/Geriatric.aspx). Score systems such as this and others used clinically as complex geriatric assessments are time-consuming and not readily available, though may be more reliable in the long run [255]. It has been shown multiple times that during therapy of elderly myeloma patients, proper determination of frailty is as important for progression-free and overall survival as molecular and genetic markers of the underlining disease [256]. Notably, all risk scoring systems have determined age as one of the most relevant risk factors for therapy failure, though it is a matter of debate what age may exactly be the best for cutoff (70, 75, or 80 years of age). An additional aspect with definite future potential is the inclusion of easily measurable predictive biomarkers in the frailty scoring system. A pioneer of this approach has been the Mayo Clinic. Their score includes the Performance Score (WHO PS), the age, and the NT-proBNP value (Table 2) [257]. Four clearly separable subgroups could be identified with widely disparate OS differences (18 to 54 months from diagnosis).

**Table 1:** The IMWG frailty determination system

A				
Variable		Hazard ratio (CI 95%)	*p*	Score
Age	Age < 75 years	1	-	0
	Age 75–80 years	1.37 (0.93–2.03)	0.114	1
	Age > 80 years	2.75 (1.81–4.18)	< 0.001	2
Charlson Index	Charlson ≤ 1	1	-	0
	Charlson ≥ 2	1.6 (1.07–2.39)	0.021	1
ADL score	ADL > 4	1	-	0
	ADL ≤ 4	1.76 (1.14–2.71)	0.01	1
IADL score	IADL > 5	1	-	0
	IADL ≤ 5	1.53 (1.03–2.27)	0.036	1
B				
Additive total score		Patient status		
**0**		Fit		
**1**		Unfit		
** ≥ **		Frail		

The table was constructed based on the article by Palumbo et al. [254]

**Table 2:** The Mayo myeloma frailty evaluation system and its influence on overall survival

A		
Variable	Value	Score
ECOG-PS	≥ 2	1
	< 2	0
Age	≥ 70 years	1
	< 70 years	0
NT-proBNP	≥ 300 ng/l	1
	< 300 ng/l	0
B		
Score	Overall survival	
0	NR	
1	58 months	
2	28 months	
3	18 months	

Each variable may score a point, creating a staging from 0 to three. This frailty score was determined to be independent of cytogenetics and the revised ISS stage of the disease. Based on Milani et al. [257]

The most important value of frailty measurement for everyday clinical practice is in the available recommendations for relevant drug dosing in the treatment of MM in older adults. Accordingly, in multiple clinical trials, evidence has been presented that elderly patients abandon therapy early and do not get its total possible value unless the anticancer drug dosing intensity has been adapted to their fitness level. Consequently, a well-designed, personalized therapy may achieve its full benefit for the patient (Table 3) [255, 258].

**Table 3:** Myeloma treatment dosing recommendations based on the patient frailty assessment, modified from Möller et al. [255], [258]

Treatment	Fit (“go-go”)	Unfit (“intermediate-go”)	Frail (“slow-go”)
Overall dosing	Standard level	Level-1	Level-2
Dexamethasone	40 mg a day, weekly	20 mg a day, weekly	8–10 mg a day, weekly
Melphalan	0.25 mg/kg, days 1–4, 4–6-week cycle	0.18 mg/kg, days 1–4, 4–6-week cycle	0.13 mg/kg, days 1–4, 4–6-week cycle
Bortezomib	1.3 mg/m^2^ twice weekly	1.3 mg/m^2^ weekly	1.0 mg/m^2^ weekly
Thalidomide	100–200 mg/day	50–100 mg/day	50 mg every other day
Lenalidomide	25 mg days 1–21 of a 28-day cycle	15 mg days 1–21 of a 28-day cycle	10 mg days 1–21 of a 28-day cycle
Ixazomib	4 mg weekly	3 mg weekly	2.3 mg weekly
Daratumumab	16 mg/kg biweekly iv or 1800 mg sc in cycles 1 + 2, in combo	16 mg/kg biweekly iv or 1800 mg sc in cycles 1 + 2	8 mg/kg initial dose, increase to 16 mg/kg or weekly 1800 mg Sc

An additional essential aspect of clinical care of elderly myeloma patients is vaccination. As infections are the leading cause of death in myeloma and the “alertness” of the aged immune system is impaired, prophylaxis of infections may be lifesaving. Regular seasonal Influenza vaccinations, *Pneumococcus* vaccination, and SARS-CoV vaccination should be done as the standard of care. Acyclovir prophylaxis is inexpensive and effective for herpes simplex and zoster and should be pursued for most patients who receive anticancer therapy.

Antibacterial prophylaxis is controversial, though the evidence is reasonably strong for initial therapy in frail patients (levofloxacin or sulfamethoxazole/trimethoprim could be used). The latter is also appropriate as *Pneumocystis* prophylaxis, especially in late-line heavily pretreated patients.

## Conclusion

MM is different from many other hematopoietic malignancies with respect to the origin of cells. It develops from terminally differentiated B cells over very long periods, possibly decades. The gradually increasing and interconnected instability of the epigenome and genome and the accumulation of mutations have common roots and are similar in many aspects during the development of aging and MM. It is easy to hypothesize that the development of MM as cancer is unusually long because it is aging associated with stochastic clone development and selection that causes it.

**Fig. 1:**
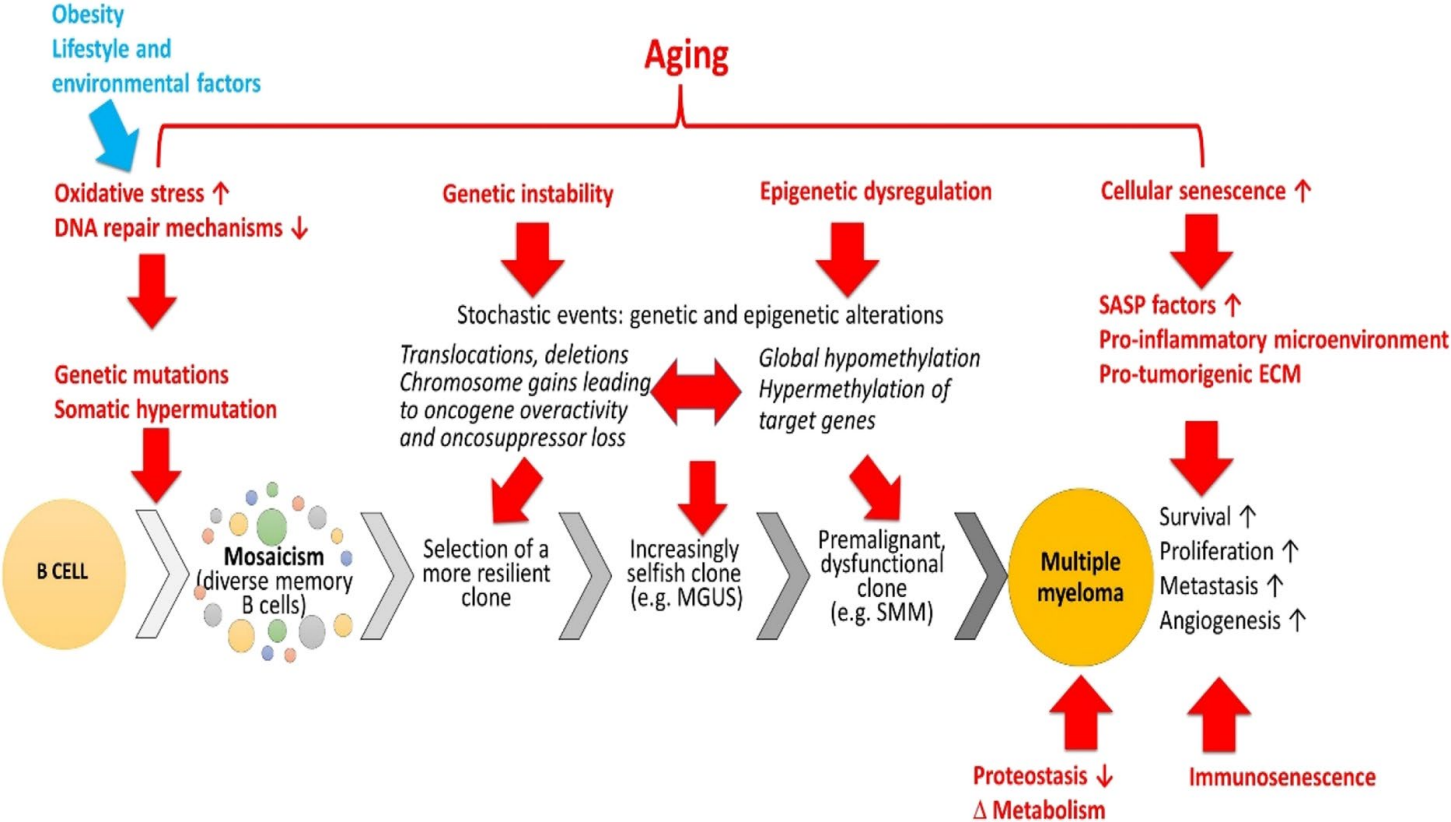
Schematic illustration of the role of fundamental cellular and molecular mechanisms of aging in the pathogenesis of multiple myeloma. The scheme highlights stages of B cell differentiation and myelomagenesis, showing how myeloma progresses from a normal plasma cell to monoclonal gammopathy of undetermined significance (MGUS) and smoldering multiple myeloma (SMM) to full blown multiple myeloma. Aging promotes the genesis of DNA damage and mutations, facilitates the selection of premalignant and malignant clones, impairs the mechanisms involved immunosurveillance and elimination of malignantly transformed cells, and exacerbates cellular and molecular mechanisms contributing to tumor cell survival, proliferation, extramedullary tumor formation, and tumor angiogenesis. Abbreviations used: SASP, senescence-associated secretory phenotype; MGUS, monoclonal gammopathies of undetermined significance; SMM, smoldering multiple myeloma

The memory B cells in germinal centers may at least potentially give rise to many plasma cells, making the cell source similar when the aged bone marrow HSCs population is already depleted, and hematopoiesis relies increasingly on progenitors. After multiple divisions, with more cellular memories of differentiation and environmental effects, a somatic cell carries much more epigenetic and genetic divergencies than a stem cell [259], [260], [261]. Aging is also characterized by mutations that lead to mosaicism, forming divergent clonal cell lines in tissues [262]. The older the body is, the fewer cells carry the original genomic blueprint of the zygote and preserve multipotency. Above a certain age, it is a matter of luck when and which clone becomes dysfunctional or malignant. In Fig. 1, we summarized aging processes and B cell maturation that act in concert in the development of MM. Aging alone is a malignancy risk, but memory B cells are especially endangered by this transformation.

Additionally, B cell development involves steps (like SHM and clonal selection) that make its genome particularly vulnerable, potentially causing an accelerated aging process in this cell type compared with others. It is stochastic which clone, a possibly transformed B cell, is activated and whether this leads to the proliferation of a truly malignant plasma cell clone. Chronic inflammation in the aging body and the general aging of the immune system as well as clonal hematopoiesis favor the development of MM by providing a nurturing environment.

In summary, the claim that aging is the most significant risk factor for cancer development is particularly striking in the case of MM. If we consider aging a multifaceted disease process, then gammopathy is one of the possible faces, and MM is the final outcome.

Treatment of the aged and polymorbid myeloma patients is incredibly challenging and puts a strain on the patient, the caregiver, and the healthcare system. When tailoring the therapy, we must consider that all organs of these elderly patients are aged and have a lower tolerance. On the other hand, myeloma in elderly patients is generally not more resistant to anticancer therapy, just one needs to apply the most adequate drug dosing and therapy intensity.
